# Erythropoietin and Friedreich Ataxia: Time for a Reappraisal?

**DOI:** 10.3389/fnins.2019.00386

**Published:** 2019-04-24

**Authors:** Sylvia Boesch, Elisabetta Indelicato

**Affiliations:** Department of Neurology, Medical University of Innsbruck, Innsbruck, Austria

**Keywords:** erythropoietin, friedreich ataxia, clinical trials, frataxin, iron, mitochondria

## Abstract

Friedreich ataxia (FRDA) is a rare neurological disorder due to deficiency of the mitochondrial protein frataxin. Frataxin deficiency results in impaired mitochondrial function and iron deposition in affected tissues. Erythropoietin (EPO) is a cytokine which was mostly known as a key regulator of erythropoiesis until cumulative evidence showed additional neurotrophic and neuroprotective properties. These features offered the rationale for advancement of EPO in clinical trials in different neurological disorders in the past years, including FRDA. Several mechanisms of action of EPO may be beneficial in FRDA. First of all, EPO exposure results in frataxin upregulation *in vitro* and *in vivo*. By promoting erythropoiesis, EPO influences iron metabolism and induces shifts in iron pool which may ameliorate conditions of free iron excess and iron accumulation. Furthermore, EPO signaling is crucial for mitochondrial gene activation and mitochondrial biogenesis. Up to date nine clinical trials investigated the effects of EPO and derivatives in FRDA. The majority of these studies had a proof-of-concept design. Considering the natural history of FRDA, all of them were too short in duration and not powered for clinical changes. However, these studies addressed significant issues in the treatment with EPO, such as (1) the challenge of the dose finding, (2) stability of frataxin up-regulation, (3) continuous versus intermittent stimulation with EPO/regimen, or (4) tissue changes after EPO exposure in humans *in vivo* (muscle biopsy, brain imaging). Despite several clinical trials in the past, no treatment is available for the treatment of FRDA. Current lines of research focus on gene therapy, frataxin replacement strategies and on regulation of key metabolic checkpoints such as NrF2. Due to potential crosstalk with all these mechanisms, interventions on the EPO pathway still represent a valuable research field. The recent development of small EPO mimetics which maintain cytoprotective properties without erythropoietic action may open a new era in EPO research for the treatment of FRDA.

## Introduction

Friedreich ataxia (FRDA) is a neurological disorder caused by homozygous intronic GAA expansions in the frataxin (*FXN*) gene (Campuzano et al., 1996). FRDA is the most common inherited ataxia in the Caucasians (Vankan, 2013). The typical presentation is with a progressive gait and limb ataxia leading to severe disabilities and loss of independent gait in most patients (Parkinson et al., 2013a). In up to 75% of patients, heart is also involved (Weidemann et al., 2012) and cardiac complications represent the first cause of death (Tsou et al., 2011; Weidemann et al., 2012). From the discovery of disease causing mutation in 1996 (Campuzano et al., 1996) clinical studies in FRDA have taken place at an impressive pace for a rare disease (Indelicato and Boesch, 2018). FRDA has been the testing field for compounds which were further developed for the treatment of other disorders (Klopstock et al., 2011; Reata Pharmaceuticals, Inc., 2017) as well as the first neurodegenerative disease in which Erythropoietin (EPO) advanced to clinical trials (Boesch et al., 2007).

Erythropoietin is a cytokine that was mostly known as the central regulator of the erythropoiesis (Jelkmann, 1992) until its receptors were identified also in non-hematopoietic tissues including the nervous system (Tan et al., 1992; Masuda et al., 1993, 1994; Morishita et al., 1997). In the past two decades, several studies in different *in vivo* and *in vitro* models have demonstrated marked neurotrophic and neuroprotective properties of EPO (Konishi et al., 1993; Morishita et al., 1997; Campana et al., 1998; Celik et al., 2002; Bianchi et al., 2004; Leist et al., 2004). Due to these findings, EPO has been investigated for the treatment of several neurologic disorders (Bianchi et al., 2004; Kollensperger et al., 2011; Lauria et al., 2015; Yao et al., 2017).

In FRDA, several mechanisms supported application of EPO as potential therapeutic compound. Indeed, EPO may influence *FXN* levels and has regulatory properties on angiogenesis, mitochondria and iron homeostasis (Sturm et al., 2005; Carraway et al., 2010; Nachbauer et al., 2011a, 2012; Plenge et al., 2012; Qin et al., 2014). EPO advanced fast to clinical pilot studies in FRDA, though its further development was hampered by challenging issues in chronic treatment as highlighted by early studies. Even though, interventions on the EPO pathway still represent a valuable therapeutic strategy. Indeed, a decade after the first EPO trial in FRDA (Boesch et al., 2007), EPO analogs are still in the pipeline (Miller et al., 2017; Alliance FsAR, 2018).

In the present paper, we review the use of EPO and derivatives in the treatment of FRDA. Firstly, we briefly summarize the key points in the pathogenesis of FRDA in order to elucidate the rationale of application of EPO and derivatives in FRDA treatment. Then, results from early clinical trials and newer evidence are summarized and critically reappraised.

## Methods

We aimed at retrieving all published basic research studies and clinical trials on EPO derivatives in FRDA. To this purpose, we carried out a targeted PubMed search using the following keywords in combination: Friedreich ataxia, Erythropoietin, clinical trials, frataxin, iron, mitochondria. The literature research was extended to pertinent references of retrieved papers. Furthermore, information on ongoing studies was collected through public channels (curefa.org, clinicaltrials.gov).

## Friedreich Ataxia

### Clinical Presentation

Friedreich ataxia typically appears in the adolescence, although later onset cases are increasingly recognized (Bhidayasiri et al., 2005). Ataxia is the cardinal feature and reflects involvement of dorsal root ganglia, posterior column of spinal cord and peripheral sensory nerves (Koeppen et al., 2009, 2017a,b; Koeppen and Mazurkiewicz, 2013). Muscle strength is initially preserved, but atrophy and paresis with an ascending pattern may develop later in the disease course (Harding, 1981; Filla et al., 1990; Durr et al., 1996; Parkinson et al., 2013a). FRDA is multisystemic disorder, with cardiomyopathy, scoliosis and pes cavus being the typically encountered non-neurological manifestations (Harding, 1981; Ackroyd et al., 1984; Filla et al., 1990; Durr et al., 1996; Schols et al., 1997; Delatycki et al., 1999; McCabe et al., 2000). Diabetes mellitus in FRDA is also more frequent than in the general population, but it is usually a later feature (Andermann et al., 1976; Harding, 1981; Ackroyd et al., 1984; Filla et al., 1990; Durr et al., 1996; Schols et al., 1997; Delatycki et al., 1999; McCabe et al., 2000).

Disease progression in FRDA can be captured by means of different clinical scales which quantify the severity of ataxic symptoms such as dysmetria, dysarthria and gait instability. The most used scales are the Scale for assessment and rating of ataxia (SARA) (Schmitz-Hubsch et al., 2006) and the Friedreich Ataxia Rating Scale (FARS) (Subramony et al., 2005). The SARA scale evaluates 8 items (gait, stance and sitting instability, upper and lower limb dysmetria, intentional tremor, dysdiadochokinesis and dysarthria) to yield a final score from 0 (no ataxia) to 40 (the most severe ataxia, bed-ridden patient). FRDA progresses steadily, but slowly. Based on natural history data, a mean deterioration of 0,77 points/year in the SARA score has been calculated (Reetz et al., 2016).

### Pathogenesis

Pathologic GAA expansions in FRDA are intronic (Campuzano et al., 1996) and result in reduced level of a structurally normal protein. GAA repeats length is tightly related to the residual transcriptional activity of *FXN* (De Michele et al., 1998; Yandim et al., 2013). Frataxin is encoded in the nucleus and is imported in the mitochondria after maturation (Koutnikova et al., 1998; Branda et al., 1999a,b; Cavadini et al., 2000). Intracellular deficiency of *FXN* is the trigger of FRDA pathology (Lynch et al., 2012; Pastore and Puccio, 2013). Accordingly, in all large clinical studies a significant inverse correlation was found between the length of the shorter GAA repeat (GAA1) and clinical milestones such age at onset, disease severity and progression rate (Reetz et al., 2015, 2016; Patel et al., 2016). The extent of this correlation is, however moderate. For example, GAA repeats accounted for only 36% of the variability of age of onset in a large European natural history study (Reetz et al., 2015).

Despite its exact biological role remains unclear, cumulative evidence demonstrated that *FXN* (1) has iron-binding properties and (2) is involved in the synthesis of iron-sulfur clusters, the prosthetic groups of several mitochondrial enzymes (Ristow et al., 2000; Huynen et al., 2001; Adinolfi et al., 2002; Gerber et al., 2003; Ramazzotti et al., 2004). Accordingly, mitochondrial iron deposition is observed in affected tissues (Koeppen et al., 2007, 2012; Koeppen and Mazurkiewicz, 2013) as well as reduced activity of the respiratory chain complexes (Bradley et al., 2000). The net effects are an increased sensitivity to oxidative stress, increased mitochondrial DNA damage, impaired mitochondrial biogenesis and bioenergetic failure (Rotig et al., 1997; Ristow et al., 2000; Tan et al., 2001; Chen et al., 2002; Shan et al., 2007; Singh et al., 2015; Jasoliya et al., 2017). The mitochondrial pathogenesis of FRDA is reflected by the peculiar clinical phenotype (ataxia, cardiomyopathy, diabetes), which resembles that of classical mitochondriopathies.

### Frataxin as a Biomarker

The identification of *FXN* mode of action in the mitochondria (Cossee et al., 1997; Bulteau et al., 2004) and the development of *FXN* measurements (Pianese et al., 2004) led to pivotal advantages in FRDA trials. Two different groups developed *FXN* assays in the early 2000. Sturm et al. (2005) used cell lysates that were separated on gel electrophoresis under non-reducing conditions and electroblotted them onto nitrocellulose membranes. Western-blot analysis was performed with a primary rabbit polyclonal antibody against mature human and mouse *FXN* (provided by Prof. Gracia Isaya, Departments of Pediatric and Adolescent Medicine and Biochemistry and Molecular Biology, Mayo Clinic College of Medicine, Rochester, MN, United States). Lynch and co-workers expanded the lateral flow immunoassay to measure *FXN* directly in buccal cells and whole blood (Deutsch et al., 2010). Their assay in buccal cells shared a similar degree of variability with previous studies conducted in lymphoblastoid cells (∼10% coefficient of variation in controls). Although some overlap between the groups was seen, *FXN* levels were inversely related to GAA repeat length and correlated directly with age of FRDA onset (Deutsch et al., 2010; Friedman et al., 2010). Currently, several assays to measure frataxin have been developed (FARA, 2019). In EPO studies, frataxin measurements were generated by ELISA or ECLIA assay (Steinkellner et al., 2010).

A relevant issue in *FXN* measurements and their meaningfulness is the understanding of *FXN* levels in different tissues. Lynch and co-workers (Lazaropoulos et al., 2015) measured *FXN* levels in buccal cells and blood in relation to disease features. Thereafter, site-directed mutant *FXN* was transfected into a human embryonic kidney cell model. GAA repeat length predicted *FXN* levels in both tissues, and *FXN* levels themselves predicted neurological ratings. These data underpinned earlier data in muscle biopsies from FRDA patients which also showed *FXN* levels that correlated GAA1 (Nachbauer et al., 2011b).

Although *FXN* measurements are a major advantage for clinical trials, some issues are unsolved. There is considerable overlap between carriers and patients (Acquaviva et al., 2008; Sacca et al., 2013); data on sensitivity to change in affected tissues in humans is still sparse (Nachbauer et al., 2011b), and the ability of compounds to increase *FXN in vivo* was strikingly diverse in several different studies (Histone deacetylase inhibitors, EPO, EPO derivatives *in vitro*; *FXN* messenger RNA versus *FXN* protein) (Libri et al., 2014; Indelicato and Boesch, 2018). In our hands *FXN* measurements were crucially dependent on the preparation of peripheral blood mononuclear cells (PBMCs), whereas buccal cells and whole blood measures had a broad range of variation in repeated measurements. Interestingly, an extra-mitochondrial *FXN* source has been newly identified in erythrocytes (Guo et al., 2018).

## Erythropoietin as Therapeutic Compound

Erythropoietin is a glycoprotein belonging to the superfamily of type I cytokine (Jelkmann, 1992). The mature EPO protein contains 165 amino acids and a carbohydrate part consisting of 4 glycans. In fetal life, EPO is produced in the liver and then, after birth, production site moves to the kidneys. Erythropoietic effects of EPO are mediated by binding to a homodimeric receptor (EPOR), member of the cytokine receptor superfamily, which is expressed on erythroid precursors in the bone marrow. EPOR activation support viability, proliferation and differentiation of erythroid lineage cells. Studies with EPO-analogs demonstrated that some cytoprotective effects of EPO are mediated by binding to receptors other than EPOR. For example, EPOR can form heterodimers with the β common receptor (ßCR), whose activation has a role in EPO mediated tissue protection. Synthetic EPO manufactured with recombinant DNA technology (rhuEPO) is currently approved to treat anemia in kidney failure, HIV infection, cancer, and chronic inflammatory disease. For these indications, dosing regimens range from 50 to 300 IU/kg three times per week (Volberding, 2000; Gilreath et al., 2014; Coronado Daza et al., 2015). RhuEPO can be typically administered subcutaneously, with a half-life up to 18 h, intravenously, with a shorter half-life ranging from 4 to 13 h (Jelkmann, 1992). Peripherally administered EPO is able to cross the blood-brain-barrier (Brines et al., 2000). Peak EPO concentration in cerebrospinal fluid occurs 1–3 h after intravenous administration and is ca. 1% of those of plasma (Brines and Cerami, 2005; Xenocostas et al., 2005). Thus, large and repeated doses need to be administered to reach an effective neuroprotection (Brines et al., 2000; Banks et al., 2004; Ehrenreich et al., 2004; Xenocostas et al., 2005).

To overcome the unwanted erythropoietic stimulation and maintain the cytoprotective effects, non-erythropoietic analogs have been synthetized via chemical modifications of EPO. These analogs include carbamylated EPO (CEPO), glutaraldehyde EPO –obtained by chemical modification of the lysine residues-, Neuro-EPO – an analog with low sialic acid content-, EPOL – a variant with low glycosylation- and neuropoietin – a less acidic EPO isoform (Leist et al., 2004; Mattio et al., 2011; Rodriguez Cruz et al., 2017; Castillo et al., 2018; Ercan et al., 2018). Recently, smaller EPO-mimetic have also been developed. For a recent review on the topic see (Ercan et al., 2018). EPO mimetics consist either of short peptides homologous to specific sequences of EPO molecule or of non-homologous compounds, peptidic or non-peptidic, which are able to bind EPO receptor and induce its activation (Ercan et al., 2018). Further advances have been made also concerning administration route. Intranasal formulations of EPO and EPO-analogs have been developed in order to avoid repeated injections or high-dose systemic administration (Genc et al., 2011; Santos-Morales et al., 2017). Indeed, the nasal route can bypass the blood brain barrier with an optimal distribution in the cerebrospinal fluid and negligible flow in the systemic circulation, thus potentially hampering unwanted side effects (Genc et al., 2011).

## Erythropoietin and FRDA: the Bench

Therapeutic options in FRDA can be broadly divided into those aiming at correcting *FXN* level and those intended to treat consequence of *FXN* deficiency (Indelicato and Boesch, 2018). Differently from most other compounds, treatment with EPO and its derivatives fall in both categories (Nachbauer et al., 2012; Indelicato and Boesch, 2018).

### Findings From Early Studies: Frataxin Upregulation

The first evidence of a potential therapeutic effect of EPO in FRDA originated from an *in vitro* study of Sturm et al. (2005). In the wave of earlier studies demonstrating that *FXN* expression can increase in response to various types of stimuli (Ghazizadeh, 2003; Sarsero et al., 2003; Turano et al., 2003), Sturm et al. (2005) tested the effect of rhuEPO in various cell types. They showed that rhuEPO significantly increased *FXN* expression in a neuronal cell line, primary human cardiac cells and in FRDA patients derived PBMCs (Sturm et al., 2005). A dose dependent RhuEPO dependent upregulation of *FXN* occurred within a short time after exposure. These results were then replicated in primary fibroblast cell cultures derived from FRDA patients by Acquaviva et al. (2008). Currently, little is known about the mechanisms underlying *FXN* upregulation upon EPO exposure. While Acquaviva et al. (2008) excluded any increase in *FXN* messenger RNA expression in their experiments, recent data from a FRDA murine model suggest that EPO-mediated *FXN* upregulation implies also a regulatory effect at a transcriptional level (Miller et al., 2017). Interestingly, CEPO, a derivative without erythropoietic properties, was shown to upregulate *FXN* similarly, to rhuEPO *in vitro* (Sturm et al., 2010). This would suggest that *FXN* upregulation may be achieved also via a pathway independent from EPOR signaling.

### Additional Mechanisms: Iron Relocation

By regulating erythropoiesis, EPO exerts a relevant influence also on iron metabolism (Camaschella et al., 2016). Under EPO treatment, mobilization from iron stores occurs in order to support heme-synthesis (Camaschella et al., 2016). The resulting serological picture resembles that of iron deficiency, with decrease in ferritin and rise in transferrin levels (Boesch et al., 2007; Siren et al., 2009; Camaschella et al., 2016). This effect has been singled out as potentially beneficial in the setting of neuroinflammation and/or neurodegeneration, since shifts in iron pool may contribute to reduce freely available iron and iron accumulation (Ehrenreich et al., 2007; Siren et al., 2009). Notably, iron chelation has been proposed as therapeutic strategy in FRDA (Pandolfo and Hausmann, 2013).

### Additional Mechanisms: Mitochondrial Regulation

Intracellular signaling activated by EPOR involves JAK/STAT, PI-3K/Akt and endothelial nitric oxide synthase (Burger et al., 2009). These pathways are crucial for mitochondrial gene activation and mitochondrial biogenesis. Evidence of protective effects of EPO via mitochondrial regulation is seen in different tissues such as the myocardium, skeletal muscle and adipocytes. Mitochondrial proliferation in murine cardiomyocytes has been demonstrated upon EPO treatment/EPO induction (Carraway et al., 2010; Hsu et al., 2013). Treatment with recombinant EPO increases skeletal muscle mitochondrial oxidative phosphorylation in healthy individuals (Plenge et al., 2012). In these studies, improved mitochondrial metabolism either preceded or exceeded the increase in hematocrit, thus ruling out a secondary response to increased blood oxygen content (Carraway et al., 2010; Plenge et al., 2012). Improved mitochondrial activity as measured by an increase of NADH/NAD ratio was documented also in PBMCs of FRDA patients upon EPO treatment (Nachbauer et al., 2011a). Very limited *in vivo* data are available. Quantification of respiratory chain and citrate synthase activity in skeletal muscle biopsies and metabolic assessment with MR spectroscopy of the calf have been carried out in FRDA patients, but no dynamics was found after a very short-term treatment with EPO (Nachbauer et al., 2012, 2013).

Erythropoietin increases mitochondrial content and oxidative respiration also in adipocytes by activating key metabolic coregulators like PGC-1α, PPARγ and PPARα (Wang et al., 2013). Notably, an independent line of research showed that PGC-1α and PPARγ are downregulated in FRDA and drove the investigation of PPARγ agonists in FRDA treatment (Marmolino et al., 2010). A modulation of mitochondrial function is also among the mechanisms of action of idebenone (Garcia-Gimenez et al., 2011), an anti-oxidant compound which has showed some beneficial effect on cardiac function in FRDA (Parkinson et al., 2013b). In at least 2 clinical studies, EPO was administered concomitantly to idebenone (Mariotti et al., 2012; Arpa et al., 2013). Though, a possible synergism between the two compounds remains speculative and deserves to be elucidated in further studies.

A significant mitochondrial modulation may not be observed under CEPO treatment due to binding to a different receptor with activation of different signaling cascade (Sato et al., 2010).

### Additional Mechanisms: Angiogenesis and Brain Glial Activation

Erythropoietin mediated neuroprotection may be achieved through a number of additional mechanisms such as stimulation of angiogenesis and glial activation (Siren et al., 2001; Sugawa et al., 2002; Brines and Cerami, 2008; Kimakova et al., 2017). Indeed, expression of EPO and its receptors in the nervous system is found also in endothelial and glial cells (Ribatti et al., 1999; Yamaji et al., 1996; Sugawa et al., 2002). Angiogenetic properties of EPO were initially advocated as a relevant mechanism of protection in models of ischemic-hypoxic brain damage (Siren et al., 2001; Wang et al., 2004) and then demonstrated in a variety of other cellular systems including pancreatic beta cells(Choi et al., 2010), retina (Watanabe et al., 2005), cardiac (van der Meer et al., 2005) and skeletal muscle (Mori et al., 2007). In FRDA patients, EPO treatment results in increased capillary density in the skeletal muscle (Nachbauer et al., 2012), thus possibly improving oxygen supply and myofiber function. Notably, EPO signaling acts synergistically with that of the powerful angiogenetic factor VEGF in hypoxic tissue (Tonges et al., 2007; Brines and Cerami, 2008), but EPO antagonizes the inflammation (Li et al., 2004) and leakiness of the blood-brain-barrier (Martinez-Estrada et al., 2003) induced by VEGF. Lastly, EPO seems to be a key glial mediator being relevant for development and/or maturation in astrocytes and oligodendroglia (Sugawa et al., 2002). It protects from glutamatergic toxicity (Morishita et al., 1997) and stimulates synaptic plasticity (Sargin et al., 2011).

## Erythropoietin and FRDA: the Bedside

Erythropoietin rapidly advanced to clinical studies in FRDA. Up to date, eight clinical studies investigated the effect of rhuEPO in FRDA patients. Additionally, one study examined safety and tolerability of CEPO for the treatment of FRDA. For an overview of the published clinical studies see Table 1.

**Table 1:** Overview of the published clinical studies on EPO and derivatives in FRDA.

Study	Study design	Treatment	No.	Study duration	Outcome measures	Results
Boesch et al., 2007	Open-label	Epoetin beta s.c., 5000 IU three times/week	12	8 weeks	Primary: stable increase of *FXN* over treatment period Secondary: SARA, levels of urinary 8-OHdG and serum peroxides	Mean 27% increase of *FXN* from baseline (range 15%–63%) in 8 out of 10 patients, with 2 non-responders. A mean 11% improvement in neurological outcome as defined by the SARA
Boesch et al., 2008	Open-label	Epoetin beta s.c., 2000 IU three times/week	8	6 months	Clinical: FARS, SARA, SF-36 Laboratory: Frataxin level, levels of urinary 8-OHdG and serum peroxides	Significant improvement in mean FARS score (8,39 points, *p* = 0.0063) and SARA score (5.42 points, *p* = 0.0045) Mean 24% (range = 0–49%) *FXN* increase over baseline.
Nachbauer et al., 2011a	Open-label	Epoetin beta s.c., 3 doses: 5000, 10000, and 30000 IU in monthly intervals	5	3 months	Primary: dose-response relationship between rhuEPO dosage and *FXN* level Secondary: Iron metabolism and mitochondrial metabolism parameters in PBMCs	No apparent dose-response relationship but allover 210% median increase (82 and 380%) of frataxin levels after 3 months. Significant increase in NADH/NAD ratio and ferritin drop, again without clear dose-escalation effect.
Nachbauer et al., 2011b, 2012	Open-label	Epoetin beta s.c., 3000 IU three times/week	7	8 weeks	SARA score, frataxin level and histologic measurement in skeletal muscle biopsies	Significant improvement in SARA score (mean 2,9 points). In skeletal muscle biopsies, 44% mean *FXN* increase and significant increase of capillary density.
Saccà et al., 2011	Open-label	Epoetin alpha s.c., once 600 IU/Kg at time zero, once 1200 IU/Kg after 3 months	10	6 months	Primary: changes in *FXN* level Secondary: iron status, ICARS, echocardiographic measurements	Slow but sustained *FXN* increase over the study period (mean 54%, range 14–144 after 6 months).Significant ferritin decrease. No change in ICARS or echocardiogram.
Mariotti et al., 2012	Randomized, placebo-controlled	Epoetin alpha i.v., 3 doses of 20000 IU every 3 weeks, 3 doses of 40000 IU every 3 weeks, 3 doses of 40000 IU every 2 weeks, OR placebo (+ idebenone 5 mg/kg/day)	16	6 months	Primary: safety and tolerability, *FXN* level Secondary: clinical measurements (SARA, 9-hole peg test, SF-36)	No significant change in *FXN* levels. No significant changes in clinical measurements
Saccà et al., 2016	Randomized, placebo-controlled	Epoetin alpha s.c., 80000, 60000, or 40000 IU rhuEPO according to body weight every 12 weeks (4 times) OR placebo	56	48 weeks	Primary: Peak oxygen uptake at cardiopulmonary exercise test Secondary: frataxin levels, echocardiography parameters, vascular reactivity, SARA, 9-hole peg test, quality of life measurements.	No significant difference concerning primary outcome and other secondary measure apart from improvement in 9-hole peg test.
Arpa et al., 2013	Open-label	Epoetin alpha 150 μg every 2 or 3 weeks (+ Idebenone and riboflavin)	9	8–56 months	Safety and tolerability, SARA, echocardiography parameters, SF-36	Improvement in SARA and echocardiographic parameters (statistically non significant)
Boesch et al., 2014	Randomized, placebo-controlled	CEPO i.v. 325 μg, three times/week OR placebo	36	2 weeks	Safety and tolerability, frataxin and *FXN* mRNA levels, levels of 8-OHdG, serum peroxides and malondialdehyde, SARA, FARS, quality of life measurements	CEPO was safe and well tolerated, no significant changes in clinical and laboratory measurements.

*No, number of patients; s.c, subcutaneous; i.v, intravenous; SARA, Scale for Assessment and Rating of Ataxia; FARS, Friedreich Ataxia Rating Scale; 8-OHdG, 8-hydroxydeoxyguanosine; SF-36, 36-Item Short Form Health Survey; ICARS, International Cooperative Ataxia Rating Scale.*

### Clinical Studies of rhuEPO in FRDA

In the first rhuEPO trial in FRDA, twelve patients were treated with 5000 IU rhuEPO administered subcutaneously three times per week for 8 weeks, with the aim of reaching a constant low-dose stimulation (Boesch et al., 2007). Frataxin levels, as measured by means of an ELISA technique, showed a mean 27% increase from baseline (range 15–63%) in 8 out of 10 patients, with 2 non-responders. A mean 11% improvement in neurological outcome as defined by the SARA scale (Schmitz-Hubsch et al., 2006) was detected in the responders. The same group carried out three other proof-of-concept open-label studies with rhuEPO (Boesch et al., 2008; Nachbauer et al., 2011a, 2012). One longer study followed-up 8 FRDA patients for 6 months under treatment with a reduced rhuEPO dose (2000 IU three times a week) (Boesch et al., 2008). Consistently with the previous study, *FXN* levels showed a mean 24% increase over baseline, again with a large intragroup variability (0–49%). An improvement in the FARS and SARA scale against baseline was also demonstrated. During treatment, a dramatic ferritin drop was observed (mean drop 85%) and phlebotomy was carried out in 4 patients. FRDA patients underwent also 1,5 T cerebral MRT at baseline and after EPO treatment. In the comparison with baseline, an increase in fractional anisotropy and axial diffusivity in cerebral hemispheres (Egger et al., 2014) as well increase of gray matter volume in pulvinar and posterior parietal cortex (Santner et al., 2014) was shown after EPO treatment.

A third study evaluated the dose-response correlation EPO-*FXN* increase by applying three single escalating rhuEPO doses (5000, 10000, and 30000 IU) in 5 FRDA patients in monthly intervals (Nachbauer et al., 2011a). Single doses were not coupled by *FXN* upregulation in the short term, while a cumulative effect with up to 210% increase in *FXN* level (IQR 82–380%; *p* = 0.03) as compared to the individual baseline level was observed after the three administrations. As previously observed, there were marked differences in the extent of *FXN* increase upon rhuEPO exposure. Also, a clear relationship between rhuEPO dose and *FXN* increase could not be shown, likely because a carry-over effect. In a further study from the same group, (Nachbauer et al., 2011b, 2012) seven FRDA patients were treated with 3000 IU rhuEPO three times weekly for 8 weeks and *FXN* measurements both in PBMCs and in skeletal muscles biopsies were carried out. Though the far lower concentration in skeletal muscle tissue, muscle *FXN* levels correlated with those of blood. Notably, while FRDA is not associated with a hematologic phenotype (Selak et al., 2011), skeletal muscle is affected by the disease. Thus, these results endorsed for the first time the use of *FXN* measurement in PBMCs as biomarker in FRDA. As previously mentioned, rhuEPO exposure resulted also in increased capillary densities in skeletal muscle after 8 weeks as compared to baseline.

Further studies were then initiated by other groups. Saccà et al. (2011) tested the effect of 2 single rhuEPO high-doses in 10 FRDA patients (600 IU/Kg and 1200 IU/Kg for a maximum dose of 40000 or 80000 IU, respectively), administered with 2 months interval. Similar to the previously described results, no short-term effect on *FXN* level was found, but a slower dose-dependent increase after 3 months, which persisted for up to 6 months (Nachbauer et al., 2011b). Mariotti et al. (2012) designed a dose-escalating trial of rhuEPO in 16 FRDA patients with a placebo-controlled design (11 patients assigned to rhuEPO, 5 to placebo). Treatment schema was based on a previous study on amyotrophic lateral sclerosis (Lauria et al., 2009) and aimed at avoiding a threatening increase in hematocrit. RhuEPO was administered every 3 weeks, starting with 20000 IU for 3 times and then with 40000 for other 3 times. Subsequently other 3 administrations of 40000 IU rhuEPO every 2 weeks followed. Differently from other trials, all study patients went on receiving idebenone treatment (5 mg/kg/day) during the trial. Although mean *FXN* levels in rhuEPO arm were higher compared to those of placebo group across the trial, this difference did not reach statistically significance, with marked variability in levels among the patients. Notably, also hematological measures did not show a marked variation. No significant differences were observed at the end of the treatment concerning SARA score.

Only one larger study assessed effect of rhuEPO on FRDA (Saccà et al., 2016). On the basis of their earlier pilot study Saccà et al. (2011), tested rhuEPO in 56 patients randomized 1:1 to receive epoetin alfa or placebo. Based on body weight, 80000, 60000, or 40000 IU rhuEPO were administered 4 times in a 12 weeks interval for a total study duration of 48 weeks. As previously observed with these extended intervals, no significant hematologic adverse events were observed, but also no significant differences concerning *FXN*. The primary endpoint of the study was the change in peak oxygen uptake (VO2 max) at the maximal exercise test, a gold standard measure of cardiorespiratory fitness; SARA score and performance at the 9-hole-peg-test, a measure of upper limb dexterity, were included as secondary endpoints. No significant difference was found in none of the study endpoints apart from performance at 9-hole-peg-test, which was significantly improved in the rhuEPO arm.

Lastly, rhuEPO was tested in a more recent small pilot study together with idebenone and riboflavin; the variability in duration of treatment exposure, as well as the combination with other compound, hampers the evaluation of clinical effects due to rhuEPO (Arpa et al., 2013).

### Limitations and Pitfalls

All rhuEPO trials conducted in FRDA had a proof-of-concept design and applied different treatment schemas both in respect of dosage, administration route and intervals. Another notable source of variability is represented by use of different essays for *FXN* testing, an assessment which can be technically challenging. Taken together these issues limit the comparability of -sometimes- strikingly different results.

Sample size in these early studies ranged from 5 to 16 patients. Only one larger randomized trial included 56 patients. All these trials were conducted prior to publication of data from the North American and European natural history studies (Reetz et al., 2015; Patel et al., 2016). These register studies provided for the first time a reliable sample size calculation for clinical trials and proved that >100 participants are required to be able to show any clinical effect in a trial on FRDA. Apart from small sample size, EPO studies included markedly heterogeneous patients collectives, both concerning age at onset and disease severity. This is of relevance, since natural history studies demonstrated that progression rate varies both depending on age at onset and disease stage (Patel et al., 2016; Reetz et al., 2016). Specifically, clinical deterioration is faster in young patients in early disease stage, namely in young, ambulatory patients. On the contrary, inclusion of wheelchair-bound patients hampers the demonstration of clinical effect because of ceiling effect in clinical progression and rating (Burk et al., 2009; Indelicato and Boesch, 2018).

Despite being underpowered to show a difference against placebo, few EPO studies reported some clinical benefits (Boesch et al., 2008; Saccà et al., 2016). These findings may be interpreted as results of shorter-term effect of EPO on muscle strength and fitness, which may provide a partial compensation for ataxia. The histological prove of EPO mediated neoangiogenesis in skeletal muscle would support this concept (Nachbauer et al., 2012).

### CEPO and FRDA

Up to date only one study has been published on the treatment of FRDA with EPO derivatives. Boesch et al. (2014) performed a multicentric proof-of-concept study to investigate safety and tolerability of CEPO for the treatment of FRDA. Thirty-six patients were enrolled and allocated in a 2:1 ratio to CEPO or placebo. A fixed dose of CEPO (325 μg) or placebo was administered intravenously three times per week for 2 weeks. CEPO was safe and well tolerated. No changes of *FXN* levels were observed in after 2 weeks compared to baseline. These results are in contrast with *in vitro* experiments showing a comparable upregulating effect of rhuEPO and CEPO (Sturm et al., 2005, 2010). The authors hypothesized that *FXN* upregulation *in vivo* may be more dependent on the erythropoietic signaling of EPO than *in vitro*, since *FXN* has a pivotal role in iron metabolism. CEPO treatment did not advance to further studies in FRDA.

## Epo Derivatives Back in the Pipeline

A decade after the first trials of rhuEPO in FRDA, research on EPO pathway is still ongoing (Miller et al., 2017; Alliance FsAR, 2018). Miller et al. (2017) investigated for the first time the *FXN* regulating effects of STS-E412 and STS-E424, smaller molecules that selectively binds the tissue-protective EPO receptor with no hematopoietic effect. The effects of STS-E412 and STS-E424 were investigated in various cellular models and in the KIKO mice model of FRDA. STS-E412 and STS-E424 treatment resulted in *FXN* increase in controls and FRDA patients derived PBMCs similar to rhuEPO. They also demonstrated that both these small agonists and rhuEPO induced *FXN* mRNA increase *in vitro*, in human cortical cells, and *in vivo*, in heart tissues of KIKO mice. Surprisingly, STS-E412 and STS-E424 treatment, but not rhuEPO, were associated to *FXN* mRNA increase also in brain tissue of KIKO mice. These findings are in agreement with early observations and add further insight in the mechanisms of action of EPO and EPO-like molecules, showing for the first time that EPO pathway implies a transcriptional regulation of *FXN*. These results, together with favorable pharmacokinetic properties (blood-brain barrier permeability, oral bioavailability, lack of erythropoietic stimulation) endorse further investigation of STS-E412 and STS-E424 for FRDA treatment.

## Conclusion and Future Perspectives

More than two decades after *FXN* gene discovery, FRDA still remain an inexorably progressive and devastating disorder. Though, clinical trials have been carried out non-stopping. All these studies have been completed before the availability of reliable clinical data for sample size calculation and validation of clinical scales for ataxia rating. Thus, despite globally negative findings, results of early trials must be interpreted with caution.

Beyond trial design pitfalls, approach selection represents a major issue in FRDA treatment. We live in the era of gene therapy, and gene/protein replacement therapy is considered the curative approach per antonomasia. While this concept holds truth for the generality of genetic disorders, some special considerations should be made about FRDA.

A number of preclinical studies support *FXN* upregulation as valuable therapeutic approach in FRDA. Histone deacetylase inhibitors, translocation of *FXN* protein via TAT-*FXN* fusion protein as wells as transfection with adenovirus carrying *FXN* gene all succeeded in ameliorating or even reversing the pathologic phenotype in different murine models of FRDA (Sandi et al., 2011; Vyas et al., 2012; Gerard et al., 2014; Perdomini et al., 2014). However, if *FXN* replacement therapy alone would be curative at the bedside remains to be proved.

Recent studies on necropsy samples from FRDA patients suggested that *FXN* deficiency might be critical in a close time window during neurodevelopment, impairing the delimitation between peripheral and central nervous system at the dorsal spinal roots (Koeppen et al., 2017b). Indeed, while is generally accepted that *FXN* deficiency produces the “first hit” in FRDA pathogenesis, the mechanisms that lead to disease progression still have to be elucidated. Progressive expansions of GAA repeats (De Biase et al., 2007) as well as accumulation of mitochondrial damage (Singh et al., 2015) have been advocated. In the light of these observations, “symptomatic” approaches, not limited to *FXN* upregulation, may still be valuable in FRDA.

Erythropoietins have been among the first compounds which have been tested in a clinical setting for the treatment of FRDA. Promising findings allowed exploiting in several studies, yet all with proof-of-concept design. While these studies did not provide sufficient evidence for a clinical application of EPO in FRDA, they highlighted some relevant challenges in the use of EPO in chronic non-hematologic disorders. However, differently from the majority of other therapeutic strategies in FRDA, EPO does not only act by regulating *FXN* expression. EPO has marked neuroprotective and neurotrophic properties and, more relevantly, it cross-talks with several other pathways (NrF2, PPARγ, SIRT), which have been implied in FRDA pathogenesis and are therefore an active field of research (Indelicato and Boesch, 2018). The multiple beneficial modes of action of EPO and its derivatives make them still valuable in FRDA treatment pipelines. To overcome erythropoietic effects and improve pharmacokinetic properties, new non-hematopoietic EPO-like compounds have been developed (Miller et al., 2017; Alliance FsAR, 2018). Preclinical proof-of-concept findings suggest that these strategies may provide a good alternative for the development of a symptomatic treatment in FRDA.

## Author Contributions

SB and EI contributed to ideation, redaction and review of the manuscript.

## Conflict of Interest Statement

The authors declare that the research was conducted in the absence of any commercial or financial relationships that could be construed as a potential conflict of interest.
